# Lung perfusion during veno-venous extracorporeal membrane oxygenation in a model of hypoxemic respiratory failure

**DOI:** 10.1186/s40635-022-00442-x

**Published:** 2022-04-25

**Authors:** Pedro Vitale Mendes, Marcelo Park, Luciano Cesar Pontes de Azevedo, Caio Cesar Araujo Morais, Marcelo Brito Passos Amato, Eduardo Leite Vieira Costa

**Affiliations:** 1 Medical Intensive Care Unit, Hospital das Clinicas HCFMUSP, University of São Paulo School of Medicine, São Paulo, SP Brazil; 2 Anesthesia, Critical Care and Pain, University of Harvard, Boston, MA USA; 3 Pulmonary Division, Instituto do Coracao (Incor), University of São Paulo School of Medicine, São Paulo, SP Brazil; 4 Research and Education Institute, Hospital Sírio-Libanês, São Paulo, Brazil

**Keywords:** Extracorporeal circulation, Acute lung injury, Acute respiratory distress syndrome, Perfusion, Ventilation–perfusion ratio, Electrical impedance

## Abstract

**Background:**

Veno-venous extracorporeal membrane oxygenation (ECMO) provides blood oxygenation and carbon dioxide removal in acute respiratory distress syndrome. However, during ECMO support, the native lungs still play an important role in gas exchange, functioning as a second oxygenator in series with ECMO. The hypoxic vasoconstriction mechanism diverts regional blood flow within the lungs away from regions with low oxygen levels, optimizing ventilation/perfusion matching. ECMO support has the potential to reduce this adaptive pulmonary response and worsen the ventilation/perfusion mismatch by raising venous oxygen partial pressure. Thus, the objective of this study was to evaluate the effect of ECMO on regional pulmonary perfusion and pulmonary hemodynamics during unilateral ventilation and posterior lung collapse.

**Methods:**

Five Agroceres pigs were instrumented, monitored and submitted to ECMO. We used the Electrical Impedance Tomography (EIT) to evaluate lung ventilation and perfusion in all protocol steps. Effects of ECMO support on pulmonary hemodynamics and perfusion involving two different scenarios of ventilation/perfusion mismatch: (1) right-lung selective intubation inducing collapse of the normal left lung and (2) dorsal lung collapse after repeated lung lavage. Data including hemodynamics, respiratory, lung perfusion/ventilation, and laboratory data over time were analyzed with a mixed generalized model using the subjects as a random factor.

**Results:**

The initiation of ECMO support provided a significant reduction in Mean Pulmonary Artery Pressure (PAPm) in both situations of ventilation/perfusion mismatch. However, distribution of lung perfusion did not change with the use of ECMO support.

**Conclusions:**

We found that the use of ECMO support with consequent increase in venous oxygen pressure induced a significant drop in PAPm with no detectable effect on regional lung perfusion in different scenarios of ventilation/perfusion mismatch.

**Supplementary Information:**

The online version contains supplementary material available at 10.1186/s40635-022-00442-x.

## Background

Veno-venous extracorporeal membrane oxygenation (ECMO) is a respiratory support characterized by blood oxygenation and carbon dioxide (CO_2_) removal by mechanically pumping the blood through a membrane, where diffusion of gases occurs. The main objective of this therapy is to allow the application of protective mechanical ventilation in patients with severe acute respiratory distress syndrome (ARDS) and, at the same time, to ensure adequate gas exchange [1–4]. However, during ECMO support, the native lungs still play an important role in gas exchange, functioning as a second oxygenator in series with ECMO. Moreover, despite ECMO support, hypoxemia may still occur in up to 20% of ARDS patients, and a stepwise approach including the optimization of residual lung function may be appropriate [5, 6]. As the lung injury gradually reverses, gas exchange by the lungs progressively improves until it becomes possible to remove the extracorporeal support.

To avoid large mismatches between ventilation and perfusion ventilation/perfusion mammals invoke a mechanism called hypoxic pulmonary vasoconstriction (HPV). It consists of vasoconstriction of arterioles that feed poorly oxygenated lung regions, redirecting blood flow to better ventilated lung areas with consequent attenuation of the ventilation/perfusion mismatch [7] For a given amount of lung collapse, typical in patients with ARDS, HPV can significantly attenuate the blood gases derangement by lowering shunt. The main modulating factor of this adaptive pulmonary vasoconstriction is the local partial pressure of oxygen through venous oxygen partial pressure (PvO_2_) and alveolar oxygen partial pressure [8–10]. Also, arterial pH and carbon dioxide partial pressure (PaCO_2_) act as cofactors, modulating HPV [11]. In this context, ECMO has the potential to weaken this adaptive pulmonary response and worsen the ventilation/perfusion mismatch by raising venous oxygen pressure (PvO_2_), with possible reversal of HPV. It has been previously described that high values of PvO_2_ with the use of a bubble oxygenator induce a complete reversal of HPV [12]. However, at the bedside, physicians usually set the ECMO support to reach normal values of PvO_2_ and near normal values of PaO_2._ Thus, the behavior of lung perfusion and lung hemodynamics with current ECMO support aiming at normal values of PvO_2_, is poorly understood. Knowing the behavior of pulmonary perfusion and hemodynamics during different scenarios of lung/perfusion mismatch may help in the treatment of hypoxemia despite ECMO support and, also, in the process of withdrawal from ECMO.

Therefore, the objective of this study was to evaluate the effect of current ECMO support and the consequent increase in PvO_2_ on pulmonary perfusion and pulmonary hemodynamics during unilateral ventilation and posterior lung collapse.

## Methods

This research was carried out in compliance with the principles of the National Institute of Health (1985) and The American Physiological Society (1995) regarding the care, handling, and use of laboratory animals, following approval by the Ethics and Research Committee of Hospital Sírio Libanês and Hospital das Clínicas HC-FMUSP (University of São Paulo School of Medicine).

### Surgical preparation of the animals

The ambient temperature was kept between 24 and 26 °C throughout the procedure. Five Agroceres pigs weighing between 30 and 35 kg were pre-anesthetized with intramuscular 0.5 mg/kg midazolam and 5 mg/kg ketamine. Subsequently, the animals were subjected to orotracheal intubation and connected to the mechanical ventilator (Servo-i, Maquet, Germany) with the following parameters: PEEP = 5 cmH_2_O, tidal volume = 8 ml/kg, FiO_2_ = 1.0, and respiratory rate titrated to an end-tidal CO_2_ pressure in the exhaled air (EtCO_2_) between 40 and 50 mmHg. All animals were monitored through a central venous catheter in the femoral vein, an arterial catheter in the femoral artery, a pulmonary artery catheter inserted through the external jugular vein, and cystostomy via a midline laparotomy. Sedation and analgesia were maintained throughout the experiment with 8 mg/kg/hour continuous intravenous (IV) propofol and 10 mcg/kg/hour continuous IV fentanyl.

The animals' central temperature was maintained between 37 and 39 °C, initially using a thermal mattress and blanket, and after the start of ECMO, through the heat generated by the system using a cardioplegia heater pump. Upon the start of ECMO, a bolus of 5000 IU of heparin, followed by a continuous infusion of 1000 IU/h, was administered to maintain systemic anticoagulation.

### ECMO cannulation

The external jugular vein was punctured using ultrasound guidance to introduce the ECMO return cannula (25 cm cannula, Edwards Lifesciences, Irvine, CA, USA). The final positioning of the cannula was checked via ultrasound-guided transhepatic visualization of the distal end of the cannula near the opening of the right atrium. Using a similar technique, the femoral vein was punctured to insert the ECMO drainage cannula (55 cm cannula, Edwards Lifesciences, Irvine, CA, USA). Both final positions of the cannulae were checked with the aid of ultrasonography to reduce blood recirculation during ECMO. We used a polymethilpenthene membrane (Biocube 4000) from Nipro Medical corporation in all experiments.

Additional file 1: Figs. S1–S3 represent a schematic drawing of the animals without ECMO circuit connected, with ECMO circuit connected but without gas flow (Gas Flow OFF), and with ECMO circuit connected with gas flow (Gas Flow ON).

### Respiratory and hemodynamic monitoring

Expired minute volume, tidal volume, respiratory rate, peak pressure, plateau pressure, positive end-expiratory pressure (PEEP), and inspired oxygen fraction were measured continuously through the mechanical ventilator. EtCO_2_ was measured using a volumetric capnograph connected to the orotracheal tube, and the animals' peripheral oxygen saturation (SpO_2_) was monitored through pulse oximetry. Arterial blood gases were monitored by collecting blood gas samples and analyzing them with the Radiometer ABL 800 equipment.

Heart rate, mean arterial pressure (MAP), central venous pressure, and mean pulmonary artery pressure (PAPm) were measured and displayed continuously through a multiparametric monitor (DX 2020, Dixtal, São Paulo, Brazil). Central venous saturation and cardiac output were measured using spectrophotometry and thermodilution, respectively (Vigilance®, Edwards Lifesciences, Irvine, CA, USA). Hypotension episodes with a MAP < 65 mmHg were treated with a 250 mL bolus of saline, up to a total of 75–100 mL/kg and with norepinephrine thereafter.

### Monitoring of pulmonary ventilation and perfusion

Pulmonary ventilation and perfusion were monitored using electrical impedance tomography (EIT) (Timpel Enlight 1800) as previously described [13, 14]. We used the first-pass indicator technique for perfusion measurements after the ventilator transitioned to continuous positive airway pressure (CPAP) mode, maintaining the same PEEP value. A contrast agent for the EIT, the indicator consisted of a 10 mL bolus of 20% NaCl, injected in approximately 2–4 s in the right atrium through the pulmonary artery catheter, 5 s after the start of CPAP. ECMO blood flow was briefly interrupted immediately before injection to avoid draining the contrast agent into the ECMO circuit.

### Calculations involved in the experiment

Pulmonary Shunt Fraction (%) = (CcO_2_ − CvO_2_) × 100 / (CcO_2_ − CaO_2_) 

CaO_2_ = 1.36 × Hb × SaO_2_ / 100 + 0.0031 × PaO_2_

CvO_2_ = 1.36 × Hb × SvO_2_ / 100 + 0.0031 × PvO_2_

CcO_2_ = 1.36 × Hb × 1 / 100 + 0.0031 × PcapO_2_

PcapO_2_ = (694 − 46) × FiO_2_ / 100 – PaCO_2_/RQ

- PaO_2_, PvO_2_ and PcapO_2_ are the arterial, venous and capillary oxygen partial pressures, respectively. PaCo_2_ corresponds to arterial partial pressure of carbon dioxide.
- FiO_2_ is the fraction of inspired oxygen.
- RQ is the respiratory quotient, assumed to be 0.8.
- CcO_2,_ CvO_2_ e CaO_2_ corresponds to capillary, venous and arterial oxygen content, respectively.

### Stages of the study procedure

**Fig. 1:**
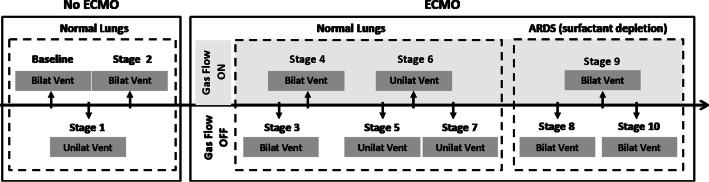
Flowchart showing the stages of the experiment. *W/O* denotes without, *ECMO* denotes extracorporeal membrane oxygenation

The study was divided into 10 stages described below (Fig. 1). These different stages aimed to create ventilation/perfusion mismatches in both normal and sick lungs to describe the degree to which the lung residual function could be worsened by the impairment of HPV caused by ECMO. Because we performed sequential interventions in all animals, we included some stages that were meant to allow the return to the previous condition after an intervention, characterizing an ON/OFF phenomenon, which supports causality even in this type of study design.

After switching from one stage to the next, the animals remained without further intervention for a period of 1 h to allow the stabilization of physiological parameters before the data collection. At the end of each stage, the distribution of ventilation and perfusion to the anterior, posterior, right, and left regions of the animals' lungs were recorded using EIT. Hemodynamic and respiratory variables, as well as extracorporeal support parameters and arterial gas analyses were recorded.

### Baseline stage: ECMO OFF + bilateral ventilation

After initial stabilization, we adjusted mechanical ventilator settings with the following parameters: PEEP = 5 cmH_2_O, tidal volume = 6 ml/kg, FiO_2_ to keep arterial saturation above 90% and respiratory rate titrated to an end-tidal CO_2_ pressure in the exhaled air (EtCO_2_) between 40 and 50 mmHg. We collected baseline data 1 h after changing the parameters.

### Stage 1: ECMO OFF + unilateral ventilation

The right main bronchus of the animal was selectively intubated, resulting in atelectasis and collapse of the left lung. The collapse was confirmed by reduction of ventilation of the atelectatic lung to less than 5–10% of the total, as visualized by EIT. Respiratory rate was set at 35 ipm and tidal volume adjusted to maintain normocapnia (PaCO_2_ between 35 and 45 mmHg) and as needed to maintain a plateau pressure of less than 30 cm H_2_O. FiO_2_ was titrated to SpO_2_ > 80–85 to simulate arterial saturation and PvO_2_ values in ARDS patients with ECMO support.

### Stage 2: ECMO OFF + bilateral ventilation

Bilateral ventilation was resumed by repositioning the orotracheal tube. Ventilation parameters were set to maintain a tidal volume of 6 ml/kg and a plateau pressure below 30 cm H_2_O. FiO_2_ was kept stable, and the respiratory rate was adjusted to maintain normocapnia.

### Stage 3: ECMO ON + gas flow OFF + bilateral ventilation

Connection of the extracorporeal circuit to the animals and start of blood flow at a rate of 500 ml/min, until the entire circuit was filled with blood from the animal. Then, the flow was progressively increased (in increments of 500 ml/min every minute) until it reached approximately 80 ml/kg. Ventilation parameters were kept stable in relation to the previous stage.

### Stage 4: ECMO ON + gas flow ON + bilateral ventilation

Start of gas flow through the extracorporeal circuit to allow gas exchange via ECMO with the objective of evaluate if initiation of ECMO support and gas flow could influence ventilation and perfusion analysis in a normal lung.

### Stage 5: ECMO ON + gas flow OFF + unilateral ventilation

Selective intubation of the right main bronchus was again performed, resulting in atelectasis and collapse of the left lung. Gas flow through the extracorporeal circuit was interrupted so that there was no gas exchange through ECMO. FiO_2_ was adjusted to keep SpO_2_ above 80–85%, and the respiratory rate was adjusted to maintain normocapnia.

### Stage 6: ECMO ON + gas flow ON + unilateral ventilation

After the data collection from the previous stage, gas flow through the extracorporeal circuit was started to allow gas exchange via ECMO with the goals to maintain SpO_2_ > 90% and normocapnia.

### Stage 7: ECMO OFF + gas flow OFF + unilateral ventilation

Condition identical to stage 5 to assess whether there was carryover effect from stage 6.

### Stage 8: ECMO ON + gas flow OFF + bilateral ventilation + Posterior Lung Collapse

The orotracheal cannula was pulled out to the trachea to allow bilateral ventilation. Subsequently, 30–40 ml/kg of warm (37–38 °C) isotonic saline solution was used for repeated lung lavages until a PaO_2_/FiO_2_ was persistently (> 10 min) below 100 mmHg as previously described [15, 16]. Ventilator parameters were PEEP = 0 cmH_2_O, tidal volume = 6 ml/kg, respiratory rate = 35 ipm, and FiO_2_ = 1.

### Stage 9: ECMO ON + gas flow ON + bilateral ventilation + Posterior Lung Collapse

The ventilation parameters were maintained. ECMO was adjusted through changes in blood and gas flow to maintain SpO_2_ > 90% and normocapnia.

### Stage 10: ECMO ON + gas flow OFF + bilateral ventilation + Posterior Lung Collapse

With parameters and goals identical to stage 8, the objective of this stage was to evaluate if the removal of ECMO support, by shutting off the gas flow, would restore lung perfusion to its previous condition (stage 8).

The objectives of each comparison among different protocol stages are better describes in the Additional file 1.

At the end of the experiment, the animals were sacrificed by deepening anesthesia and subsequent administration of intravenous potassium chloride (66 mg/kg).

### Statistical analysis

Our main outcome was lung perfusion changes between different study stages. Given the lack of published data on this outcome, we did not perform a sample size calculation. We chose to study five animals in this proof-of-concept study with granular and good quality data. Continuous data are presented as medians [interquartile range]. Continuous data of hemodynamic, respiratory, lung ventilation/perfusion, and laboratory data over time were analyzed using a mixed generalized model with the subjects as a random factor. *P* < 0.05 was considered statistically significant. R-Free statistical software (Vienna, Austria, 2009) was used for analysis and graph construction.

## Results

We included five animals in the study and all data regarding lung ventilation and perfusion were available for analysis. We were able to induce lung collapse with selective intubation of the right lung, which led to near zero ventilation to the left lung. Perfusion to the collapsed lung was significantly reduced. Left lung collapse was also associated with a significant increase in mean pulmonary arterial pressure and in the fraction of pulmonary shunt (Additional file 1: Fig. S4). The initiation of blood circulation throughout the ECMO circuit did not affect the reduction in the left lung ventilation or perfusion with unilateral lung collapse (Additional file 1: Fig. S6).

**Fig. 2:**
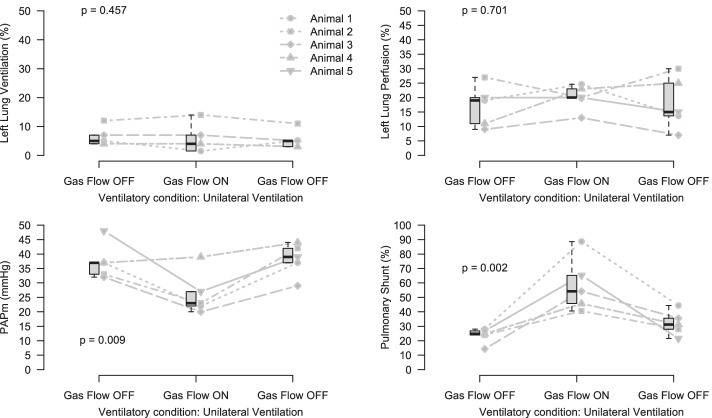
Left lung ventilation in % of lung ventilation (top left); left lung perfusion in % of pulmonary blood flow (top right); mean pulmonary artery pressure (bottom left); pulmonary shunt (bottom right). Gas flow OFF denotes no ECMO support due to the absence of gas flow. Gas flow ON denotes the initiation of ECMO support

Despite the increase in PvO_2_ after the start of ECMO, the left lung perfusion did not change (Fig. 2). Pulmonary shunt, however, increased significantly and was associated with a reduction in PAPm without any change in cardiac output. Data regarding hemodynamics and respiratory variables during stages 5, 6, and 7 are described in Table 1.

**Table 1:** Comparison between stages 5 (S5), 6 (S6) and 7 (S7), that is, unilateral lung ventilation without gas flow (Stages 5 and 7) and with gas flow (Stage 6)

	S5: Unilateral ventilation ECMO + Gas Flow OFF	S6: Unilateral ventilation ECMO + Gas Flow ON	S7: Unilateral ventilation ECMO + Gas OFF	*P* value
*Respiratory variables*				
Respiratory Rate (ipm)	35 [35, 35]	35 [35, 35]	35 [35, 35]	0.409
Tidal volume (ml)	160 [152, 175]	150 [149, 155]	170 [164, 200]	0.456
Plato pressure (cmH_2_O)	24 [19, 31]	23 [22, 26]	32 [25, 34]	0.045
FiO_2_ (%)	30 [25, 35]	35 [30, 50]	35 [35, 60]	0.378
PEEP (cmH_2_O)	5 [5]	5 [5]	5 [5]	1.000
PaO_2_ (mmHg)	61,8 [56.7, 68.0]	77.3 [68, 95]	56.1 [53.7, 59.2]	0.011
PaCO_2_ (mmHg)	43.3 [38.5, 47.1]	42.7 [39.7, 45.3}	45.2 [39.5, 48,0]	0.240
SaO_2_ (%)	85.7 [84.9, 88.8]	92.4 [91.8, 94.5]	80.2 [76.0, 87.1]	0.004
PvO_2_ (mmHg)	29 [25.3, 30.4]	56.1 [51.5, 63.7]	27.4 [25.8, 29.7]	0.002
SvO_2_ (%)	45 [43,46]	84 [83,88]	42 [40,58]	0.004
*Hemodynamic variables*				
Heart rate (bpm)	122 [114, 134]	117 [95, 120]	136 [123, 142]	0.122
MAP (mmHg)	105 [101, 109]	103 [86, 109]	104 [83, 112]	0.437
MPAP (mmHg)	37 [33,37]	23 [22,27]	39 [37,43]	0.010
CVP (mmHg)	13 [9, 13]	11 [11, 15]	10 [7, 13]	0.525
Wedge pressure (mmHg)	13 [12, 13]	9 [8, 10]	10 [9, 13]	0.019
Cardiac output (l/min)	4.6 [4.3, 5.7]	4.3 [3.5, 6.8]	3.9 [3.8, 5.7]	0.160
Metabolic variables				
Temperature (^o^C)	38 [37.2, 38.2]	37.9 [36.8, 38.4]	38 [37.2, 38.4]	0.254
pHa	7.39 [7.17, 7.39]	7.40 [7.39, 7.40]	7.35 [7.27, 7.37]	0.347
BE	− 0.2 [− 10.4, 1.3]	0.1 9–3.6, 1.2]	− 1.9 [− 9.5, 1.5]	0.697
Lactate (mg/dl)	8 [5, 14]	6 [5, 10]	9 [5, 10]	0.564
Hemoglobin (g/dl)	8.9 [8.7, 9.7]	8.4 [8.2, 8.5]	9.5 [7.6, 10.2]	0.412

SvO_2_ denotes venous oxygen saturation acquired in the Pulmonary Arterial Catheter; PaO_2_ and PvO_2_ denotes arterial and venous oxygen partial pressure, respectively; PaCO_2_ denotes arterial carbon dioxide partial pressure; SaO_2_ denotes arterial oxygen saturation; MAP denotes mean arterial pressure; MPAP denotes mean pulmonary arterial pressure; CVP denotes central venous pressure; and BE denotes Base Excess

**Fig. 3:**
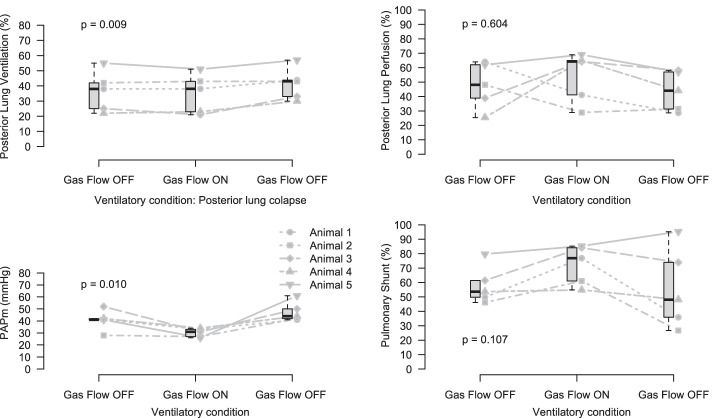
Posterior lung ventilation in % of lung ventilation (top left); posterior lung perfusion in % of pulmonary blood flow (top right); mean pulmonary artery pressure (bottom left); pulmonary shunt (bottom right). Gas flow OFF denotes no ECMO support due the absence of gas flow. Gas flow ON denotes the initiation of ECMO support

After lavage-induced lung injury and surfactant depletion (stages 8 through 10), we noticed a reduction in ventilation to dorsal regions secondary to lung collapse. Although this finding remained consistent throughout the 3 stages, we found an increase in posterior lung ventilation from Stage 8 to 10 due to progressive natural lung recruitment (Fig. 3). ECMO with Gas Flow ON increased the PvO_2_ and reduced the PAPm without any change in Cardiac Output (Fig. 3). In this scenario, we did not find a change in the pulmonary shunt or lung perfusion (Fig. 3). Data regarding hemodynamic and respiratory variables during stages 8, 9 and 10 are described in Table 2.

**Table 2:** Comparison between stages 8 (S8), 9 (S9) and 10 (S10), that is, variables with and without ECMO support after lung lavage

	S8: After surfactant depletion ECMO + Gas Flow OFF	S9: After surfactant depletion ECMO + Gas Flow ON	S10:After surfactant depletion ECMO + Gas Flow OFF	*P* value
*Respiratory variables*				
Respiratory rate (ipm)	35 [35, 35]	35 [35, 35]	35 [35, 35]	1.000
Tidal volume (ml)	160 [145, 173]	150 [134, 173]	147 [144, 178]	0.031
Plato pressure (cmH_2_O)	23 [20, 26]	24 [21, 26]	23 [21,2 6]	0.656
FiO_2_ (%)	100 [100, 100]	100 [100, 100]	30 [30, 35]	0.001
PEEP (cmH_2_O)	0 [0, 0]	0 [0, 0]	0 [0, 0]	1.000
PaO_2_ (mmHg)	50.3 [48.7, 53.5]	72.7 [67.9, 108.0]	50.5 [36.2, 54.1]	0.002
PaCO_2_ (mmHg)	62.5 [40.0, 63.8]	52.1 [40.9, 53.5]	67.9 [52.3, 68]	0.067
PvO_2_ (mmHg)	34.5 [25.7, 34.9]	63.8 [57.1, 65.3]	27.7 [24, 31.9]	0.001
SaO_2_ (%)	71.7 [60.6,73]	91.4 [89.6,96.1]	66.6 [33.0,80.2]	0.006
SvO2 (%)	48 [47,50]	81 [79,90]	49 [41,63]	0.002
*Hemodynamic variables*				
Heart rate (bpm)	151 [147, 154]	123 [106, 128]	155 [137, 175]	0.051
MAP (mmHg)	78 [76, 94]	82 [75, 104]	97 [77, 99]	0.622
MPAP (mmHg)	41 [41, 42]	31 [27, 33]	44 [42, 50]	0.010
CVP (mmHg)	10 [8, 12]	14 [11, 17]	15 [11, 16]	0.010
Wedge pressure (mmHg)	10 [8, 10]	9 [9, 14]	10 [8, 14]	0.639
Cardiac Output (l/min)	6.7 [6.2, 9.3]	6,8 [4.9, 20]	6.5 [6.5, 6.7]	0.369
*Metabolic variables*				
Temperature (^o^C)	38 [37.2, 38.2]	37.9 [37.0, 38.0]	37.9 [36.9, 38]	0.282
pHa	7.22 [7.19, 7.25]	7.17 [7.06, 7.19]	7.16 [6.96, 7.19]	0.171
BE	− 1.9 [− 8.6, − 1.8]	− 3.0 [− 17, − 1.7]	− 16.5 [− 18.5, − 1.6]	0.295
Lactate (mg/dl)	12 [11, 12]	8 [8, 16]	8 [7,23]	0.445
Hemoglobin (mg/dl)	10 [8.5, 10.1]	8.6 [7.6, 10]	9.2 [8.4, 10.1]	0.084

SvO_2_ denotes venous oxygen saturation acquired in the Pulmonary Arterial Catheter; PaO_2_ and PvO_2_ denotes arterial and venous oxygen partial pressure, respectively; PaCO_2_ denotes arterial carbon dioxide partial pressure; SaO_2_ denotes arterial oxygen saturation; MAP denotes mean arterial pressure; MPAP denotes mean pulmonary arterial pressure; CVP denotes central venous pressure; and BE denotes Base Excess

## Discussion

The main findings of this study are summarized as follows: the use of ECMO support induced a significant drop in PAPm in different scenarios of ventilation/perfusion mismatch. Pulmonary shunt increased during ECMO support in unilateral lung ventilation. We were unable to demonstrate that ECMO support affected lung perfusion distribution.

In the first stages of the present study (without ECMO), we showed that left lung perfusion was significantly reduced after complete left lung atelectasis (Additional file 1: Fig. S4). Although with great biological variability, the HPV reflex has been shown to restore to some extent adequate lung ventilation/perfusion ratios as a compensatory mechanism [10]. We demonstrated an average reduction in perfusion to the atelectatic lung of 40%, a finding compatible with other descriptions in the literature [12, 13]. Redirection of perfusion to the right lung increased PAPm but was not enough to entirely avoid the increase in pulmonary shunt. Despite total collapse of one lung, shunt values averaged below 20%, reflecting the importance of this mechanism for adequate gas exchange under pathologic conditions.

The main determinants of HPV occurrence are alveolar partial pressure of oxygen and PvO_2_ [17]. By returning highly oxygenated blood to the right circulation, ECMO can significantly increase PvO_2_ [18], depending on the ECMO settings, especially blood flow and oxygen fraction of the sweep gas. For example, ECMO settings designed to increase PvO_2_ to above 100 mmHg have been shown to entirely revert HPV in mongrel dogs with left lung atelectasis [12]. Unlike previous studies, we designed our experiments to target arterial oxygenation at clinically relevant values with the aim to investigate whether the PvO_2_ values reached in these conditions were high enough to significantly impair HPV. In addition, another major contribution of our study to what they have already shown is the inclusion of a more clinically relevant scenario in which we induced dorsal lung collapse, mimicking dorsal atelectasis common during general anesthesia or in patients with ARDS.

With left lung atelectasis, median PvO_2_ values increased from 29 to 56 mmHg after the start of ECMO support. With ECMO, we found evidence to support the occurrence of at least some inhibition of HPV although we failed to demonstrate a significant restoration of perfusion to the atelectatic lung. There was a consistent reduction in PAPm together with an increase in pulmonary shunt during unilateral lung ventilation and regional lung hypoxia (Fig. 2), whereas PAPm reduced with no significant changes in pulmonary shunt with the posterior lung collapse that ensued after lung injury (Fig. 3). The progressive posterior lung recruitment over time may have attenuated changes in pulmonary shunt in the latter scenario. On the other hand, PAPm remained stable after initiation of ECMO support during bilateral ventilation in a situation without hypoxia (Additional file 1: Fig. S5). These findings suggest that HPV inhibition occurred not only in the region of interest being studied (either left lung or dorsal atelectasis) but also in other parts of the lungs. Alternatively, it is possible that EIT lacked sensitivity to detect changes in perfusion that might have occurred. In another experimental study, EIT was able to detect restoration of perfusion to atelectatic lungs after the infusion of intravenous nitroprusside [13]. This finding is suggestive that the regional inhibition of HPV caused by the increase in PvO_2_ was not as large as that induced by intravenous nitric oxide donors, if at all. It is interesting to notice that our findings of lack of perfusion redistribution with normal ranges of PvO_2_ are compatible with a previous report in mongrel dogs monitored using electromagnetic probes placed around the left pulmonary artery that failed to show perfusion changes to the atelectatic lung when PvO_2_ was below 100 mmHg.

The changes in shunt and PAPm cannot be overlooked. The almost 30% reduction in PAPm after the start of ECMO can certainly be of value in patients with increased right ventricle afterload, especially in those with acute cor pulmonale. Conversely, it is important to emphasize that this improvement in hemodynamics comes at a cost in terms of shunt, which can be clinically relevant in patients with arterial hypoxemia despite the ECMO support. In addition, this increase in shunt should be considered when assessing residual lung function in the scenario of ECMO withdrawal. It is possible that fine tuning sweep gas and blood flow may reduce pulmonary shunt and help in the process of weaning from ECMO.

Finally, we reasoned it would be important to reproduce previous findings with current ECMO technology, more biocompatible as compared to bubble oxygenators, which have fallen out of use. The improved biocompatibility activates to a less extent the inflammation cascade, which can influence the strength of HPV. In our study, we were able to confirm Domino et al. [12] findings with unilateral ventilation using current technology of ECMO support and expanded their results to a more clinically relevant scenario of posterior lung collapse, mimicking patients posterior lung collapse.

Our study has several limitations. First, the small number of animals may lead to a type II error. We should be able, however, to detect large changes in lung perfusion, which did not occur. Moreover, two previous studies with different strategies to evaluate lung perfusion were able to detect significant changes in pulmonary perfusion with a similar number of experiments [12, 13]. Second, EIT has not been used to estimate perfusion during ECMO support. For this reason, we performed the perfusion measurements during brief (seconds) pauses in ECMO blood flow, which could have affected HPV. Kinetics of HPV, however, are well-described and the effect usually takes many minutes to subside [19]. Also, we chose to change the mechanical ventilator FiO_2_ during the experiment to assure a low PvO_2_ throughout the experiment. It is debatable if concomitantly changing the mechanical ventilator FiO_2_ and ECMO parameters may influence the results. However, alveolar partial pressure of oxygen is close to zero in collapsed regions and changes in FiO_2_ between stages of interest were small and nonsignificant. Finally, we were able to induce posterior lung collapse with repeated pulmonary lung lavage with normal saline. However, this is not an appropriate model of ARDS, since systemic inflammation, which is crucial in pathophysiology of ARDS, was not present. Possibly, the absence of systemic inflammatory response may have influenced the occurrence of HPV, affecting our results.

## Conclusions

We found that the use of ECMO support with consequent increase in PvO_2_ in different scenarios of ventilation/perfusion mismatch induces a significant drop in PAPm with a possible increase in pulmonary shunt. However, ECMO support did not affect the distribution of lung perfusion in conditions of ventilation/perfusion mismatch.

## Supplementary Information

**Additional file 1: Figure S1.** Schematic drawing representing experimental model without ECMO canulation. Deoxygenated blood return from peripheral compartment to the right side of the heart. The heart pumps the blood to the native lung in which gas exchange occurs. The oxygenated blood return to the left side of the heart and is posteriorly pumped to the peripheral compartment. **Figure S2.** Schematic drawing representing experimental model after ECMO canulation but without gas flow (Gas Flow OFF). Deoxygenated blood returning from peripheral compartment pass partially through ECMO system. However, since there is no gas flow in the circuit, the blood still returns to the circulation deoxygenated and gas exchange still occurs exclusively on native lungs. **Figure S3.** Schematic drawing representing experimental model after ECMO canulation and with gas flow (Gas Flow ON). Deoxygenated blood returning from peripheral compartment pass partially through ECMO system. Gas exchange occurs in the ECMO system and blood returns oxygenated to the right side of the heart. The heart pumps the blood to the native lung in which gas exchange also occurs (acting as two oxygenators in series). The oxygenated blood return to the left side of the heart and is posteriorly pumped to the peripheral compartment. **Figure S4. **(A) : Lung ventilation before and after selective intubation with left lung atelectasis without ECMO circuit. Left lung ventilation was reduced to near zero values and returned to normal values after returning bilateral ventilation. (B): Left lung perfusion before and after unilateral ventilation with left lung atelectasis without ECMO circuit. Left lung perfusion significantly decreased after selective intubation and returned to previous values after returning to bilateral ventilation. (C): Pulmonary shunt before and after unilateral ventilation with left lung atelectasis without ECMO circuit. Pulmonary shunt significantly increased after selective intubation and returned to previous values after returning to bilateral ventilation. (E): PAPm = Mean Pulmonary Artery Pressure. PAPm before and after unilateral ventilation with left lung atelectasis without ECMO circuit. PAPm significantly increased after selective intubation and returned to previous values after returning to bilateral ventilation. Data was not homogenous to all animals. **Table S1.** Comparison between stages Baseline, Stage 1 and Stage 2. Before installation of ECMO circuit in two different scenarios: Bilateral ventilation and unilateral ventilation. PAC SvO2 denotes venous oxygen saturation acquired in the Pulmonary Arterial Catheter; PaO2 and PvO2 denotes arterial and venous oxygen partial pressure, respectively; PaCO2 denotes arterial carbon dioxide partial pressure; SaO2 denotes arterial oxygen saturation and BE denotes Base Excess. **Table S2.** Comparison between stages 3 (S3) and 4 (S4). Variables without (Gas Flow OFF) and with ECMO support (Gas Flow ON). PAC SvO2 denotes venous oxygen saturation acquired in the Pulmonary Arterial Catheter; PaO2 and PvO2 denotes arterial and venous oxygen partial pressure, respectively; PaCO2 denotes arterial carbon dioxide partial pressure; SaO2 denotes arterial oxygen saturation and BE denotes Base Excess. **Figure S5.** (A):PvO2=Venous Oxygen partial pressure. PvO2 significantly increased after initiation of ECMO support. (B): PAPm=Mean Pulmonary Artery Pressure. PAPm did not change after initiation of ECMO support during bilateral ventilation and no previous hypoxemia. (C): Left/Right Lung perfusion before and after initiation of ECMO support during bilateral ventilation. ECMO support by itself did not promote any variation on lung perfusion. (D): Anterior/Posterior lung perfusion before and after initiation of ECMO support during bilateral ventilation. ECMO support by itself did not promote any variation on lung perfusion. **Table S3.** Comparison between stages 3 (S3) and 5 (S5). Bilateral ventilation (S3) and Unilateral Ventilation (S5) after blood circulation through ECMO circuit. PAC SvO2 denotes venous oxygen saturation acquired in the Pulmonary Arterial Catheter; PaO2 and PvO2 denotes arterial and venous oxygen partial pressure, respectively; PaCO2 denotes arterial carbon dioxide partial pressure; SaO2 denotes arterial oxygen saturation and BE denotes Base Excess. **Figure S6.** (A): Left lung ventilation before and after selective intubation and left lung collapse with blood flow through ECMO circuit. There was a significant reduction in left lung ventilation. (B): PvO2=Venous Oxygen partial pressure. PvO2 remained stable after selective lung ventilation and left lung collapse with blood flow through ECMO circuit. (C): Left lung perfusion before and after selective intubation and left lung collapse with blood flow through ECMO circuit. Blood flow through ECMO did not affect the capacity of the EIT to detect the reduction in left lung perfusion.

## Data Availability

Data are available by contacting the corresponding author.
