# Supplementary material for: Lung perfusion during veno-venous extracorporeal membrane oxygenation in a model of hypoxemic respiratory failure
Source: Intensive Care Med Exp. 2022 Apr 25;10:15. doi: 10.1186/s40635-022-00442-x (PMC9038965; doi:10.1186/s40635-022-00442-x)
Supplement: Supplementary file 1 — Additional file 1: Figure S1. Schematic drawing representing experimental model without ECMO canulation. Deoxygenated blood return from peripheral compartment to the right side of the heart. The heart pumps the blood to the native lung in which gas exchange occurs. The oxygenated blood return to the left side of the heart and is posteriorly pumped to the peripheral compartment. Figure S2. Schematic drawing representing experimental model after ECMO canulation but without gas flow (Gas Flow OFF). Deoxygenated blood returning from peripheral compartment pass partially through ECMO system. However, since there is no gas flow in the circuit, the blood still returns to the circulation deoxygenated and gas exchange still occurs exclusively on native lungs. Figure S3. Schematic drawing representing experimental model after ECMO canulation and with gas flow (Gas Flow ON). Deoxygenated blood returning from peripheral compartment pass partially through ECMO system. Gas exchange occurs in the ECMO system and blood returns oxygenated to the right side of the heart. The heart pumps the blood to the native lung in which gas exchange also occurs (acting as two oxygenators in series). The oxygenated blood return to the left side of the heart and is posteriorly pumped to the peripheral compartment. Figure S4. (A) : Lung ventilation before and after selective intubation with left lung atelectasis without ECMO circuit. Left lung ventilation was reduced to near zero values and returned to normal values after returning bilateral ventilation. (B): Left lung perfusion before and after unilateral ventilation with left lung atelectasis without ECMO circuit. Left lung perfusion significantly decreased after selective intubation and returned to previous values after returning to bilateral ventilation. (C): Pulmonary shunt before and after unilateral ventilation with left lung atelectasis without ECMO circuit. Pulmonary shunt significantly increased after selective intubation and returned to p [file 40635_2022_442_MOESM1_ESM.docx]

**Supplementary Appendix**

This appendix formed part of the original manuscript and give additional information about the work.

Additional information:

- Schematic Drawing of ECMO support
  - Figure 1s: Animal without ECMO circuit. Page 2
  - Figure 2s: Animal with ECMO circuit but no gas flow Page 2
  - Figure 3s: Animal with ECMO circuit and gas flow Page 2
- Stages of the protocol and intended objectives
  - Comparison Stages: Baseline – Stage 1 – Stage 2 Page 3
  - Comparison Stages: Stage 3 – Stage 4 Page 7
  - Comparison Stages: Stage 3 – Stage 5 Page 10
  - Comparison Stages: Stage 5 – Stage 6 – Stage 7 Original Manuscript
  - Comparison Stages: Stage 8 – Stage 9 – Stage 10 Original Manuscript

The figures below represent a schematic drawing of ECMO support.


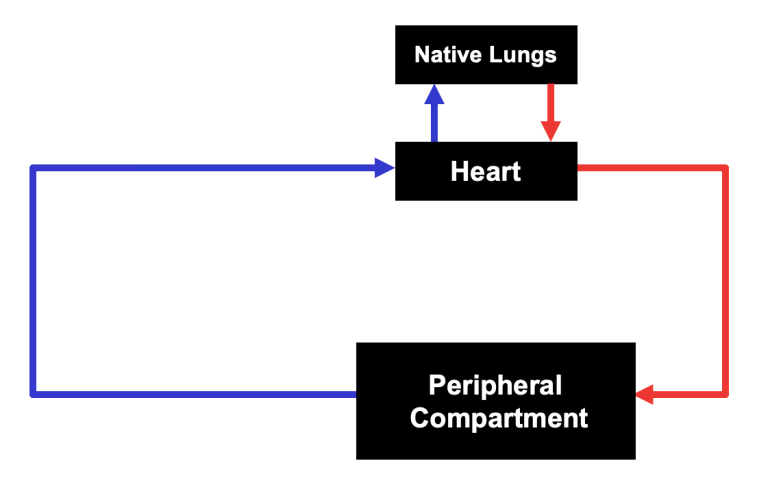
Figure 1s.: Schematic drawing representing experimental model without ECMO canulation. Deoxygenated blood return from peripheral compartment to the right side of the heart. The heart pumps the blood to the native lung in which gas exchange occurs. The oxygenated blood return to the left side of the heart and is posteriorly pumped to the peripheral compartment.


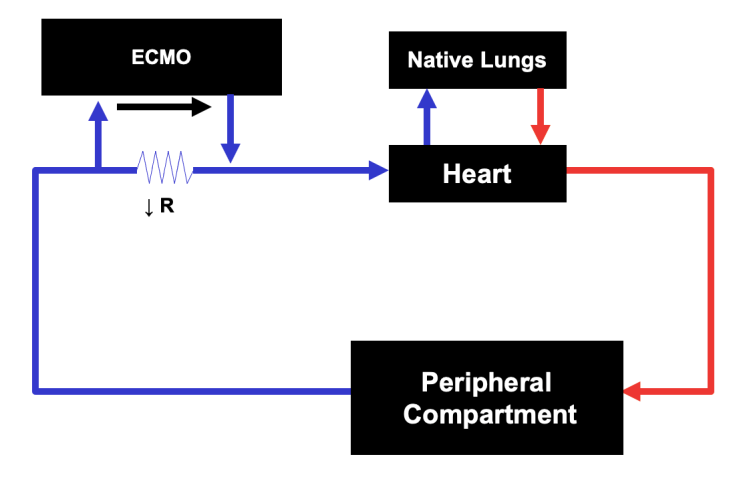


Figure 2s.: Schematic drawing representing experimental model after ECMO canulation but without gas flow (Gas Flow OFF). Deoxygenated blood returning from peripheral compartment pass partially through ECMO system. However, since there is no gas flow in the circuit, the blood still returns to the circulation deoxygenated and gas exchange still occurs exclusively on native lungs.


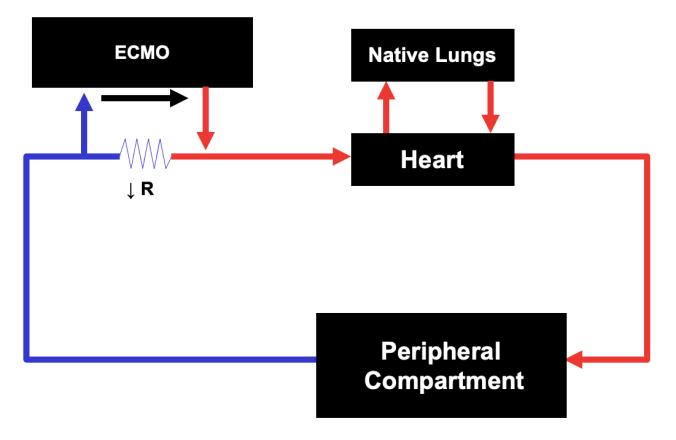
Figure 3s.: Schematic drawing representing experimental model after ECMO canulation and with gas flow (Gas Flow ON). Deoxygenated blood returning from peripheral compartment pass partially through ECMO system. Gas exchange occurs in the ECMO system and blood returns oxygenated to the right side of the heart. The heart pumps the blood to the native lung in which gas exchange also occurs (acting as two oxygenators in series) . The oxygenated blood return to the left side of the heart and is posteriorly pumped to the peripheral compartment.

**Comparison: Baseline -> Stage 1 -> Stage 2**

Objective: To test the proposed model and evaluate the behavior of ventilation, perfusion and hemodynamic parameters with selective intubation.

To check if selective intubation is capable of reducing ventilation and perfusion in the collapsed lung, as well as to check if EIT is able to detect this behavior.

|  | **Baseline**  **(Bilateral Ventilation )** | **Stage 1**  **(Unilateral Ventilation)** | **Stage 2**  **(Bilateral Ventilation )** | **P value** |
| --- | --- | --- | --- | --- |
| Respiratory Rate (ipm) | 20 [18,32] | 35 [35,35] | 22 [20,30] | **0.004** |
| Tidal Volume (ml) | 260 [255,270] | 170 [165,180] | 260 [238,270] | **<0.001** |
| Plato Pressure (cmH_2_O) | 17 [15,20] | 22 [20,23] | 17 [17,18] | **0.003** |
| FiO2 (%) | 30 [25,30] | 25 [21,30] | 30 [25,30] | 0.409 |
| PEEP (cmH_2_O) | 5[5,5] | 5[5,5] | 5[5,5] |  |
|  |  |  |  |  |
| Heart Rate (bpm) | 73 [72,80] | 70 [67,77] | 74 [69,74] | 0.612 |
| Mean Arterial Pressure  (mmHg) | 120 [118,123] | 127 [112,132] | 133 [131,134] | **0.040** |
| Mean Pulmonary Artery Pressure  (mmHg) | 28 [28,30] | 35 [33,39] | 27 [26,31] | **0.033** |
| Central Venous Pressure  (mmHg) | 12 [10,16] | 14 [11,14] | 14 [12,14] | 0.844 |
| Pulmonary Artery Occlusion Pressure  (mmHg) | 12 [10,14] | 15 [12,17] | 14 [13,15] | 0.435 |
| Cardiac Output (l/min) | 4.1 [3.2,4.2] | 3.4 [2.8,3.6] | 3.9 [3.3,4.6] | 0.452 |
|  |  |  |  |  |
| Temperature (^o^C) | 36.5 [35.1,37.1] | 38.1 [36.5,38.5] | 37.9 [37,38.2] | **0.002** |
| PAC SvO2 (%) | 80 [75,81] | 53 [52,59] | 60 [56,63] | **<0.001** |
| pHa | 7.33 [7.31,7.42] | 7.44 [7.25,7.46] | 7.40 [7.10,7.42] | 0.177 |
| PaO2 (mmHg) | 108 [97.5,108] | 59.8 [56.3,63.9] | 76.3 [76.2,93.7] | **0.036** |
| PaCO2 (mmHg) | 37.8 [35.5,49.3] | 38.2 [34.4,44] | 41.9 [35.3,43.1] | 0.777 |
| PvO2 (mmHg) | 47.7 [44.0,50,9] | 36.2 [29,36.7] | 44.5 [38.5,46.5] | **0.002** |
| BE | -3.1 [-9.62,3.1] | 5.5 [3.25,7.05] | 3.5 [-3.2,6.4] | **0.002** |
| SaO2 (%) | 97.3 [96.6,97.6] | 87.9 [86.1,89.6] | 92.8 [92.5,96] | **0.028** |
| Lactate (mg/dl) | 9 [7,9] | 6 [5,8] | 6 [5,7] | **0.017** |
| Hemoglobin (mg/dl) | 10.5 [10.2,10.7] | 11.0 [10.9,11.2] | 11.1 [10.9,11,2] | **0.046** |

Table 1s: Comparison between stages Baseline, Stage 1 and Stage 2. Before installation of ECMO circuit in two different scenarios: Bilateral ventilation and unilateral ventilation. PAC SvO2 denotes venous oxygen saturation acquired in the Pulmonary Arterial Catheter; PaO2 and PvO2 denotes arterial and venous oxygen partial pressure, respectively; PaCO2 denotes arterial carbon dioxide partial pressure; SaO2 denotes arterial oxygen saturation and BE denotes Base Excess.

Figure 4s(A) : Lung ventilation before and after selective intubation with left lung atelectasis without ECMO circuit. Left lung ventilation was reduced to near zero values and returned to normal values after returning bilateral ventilation.

Figure 4s (B): Left lung perfusion before and after unilateral ventilation with left lung atelectasis without ECMO circuit. Left lung perfusion significantly decreased after selective intubation and returned to previous values after returning to bilateral ventilation.

.

Figure 4s (C): Pulmonary shunt before and after unilateral ventilation with left lung atelectasis without ECMO circuit. Pulmonary shunt significantly increased after selective intubation and returned to previous values after returning to bilateral ventilation.

Figure 4s (E): PAPm = Mean Pulmonary Artery Pressure. PAPm before and after unilateral ventilation with left lung atelectasis without ECMO circuit. PAPm significantly increased after selective intubation and returned to previous values after returning to bilateral ventilation. Data was not homogenous to all animals.

Comparison: Stage 3 -> Stage 4

Objective: To evaluate whether starting extracorporeal support is capable of increasing PvO2 (the main determinant in inducing pulmonary arterial vasoconstriction), and whether the support alone could induce changes in pulmonary ventilation and perfusion.

|  | **S3-Bilateral Ventilation**  **ECMO + Gas Flow OFF** | **S4-Bilateral Ventilation**  **ECMO + Gas Flow ON** | **P Value** |
| --- | --- | --- | --- |
| Respiratory Rate (ipm) | 22 [20,25] | 25 [18,35] | 0.877 |
| Tidal Volume (ml) | 240 [238,250] | 229 [210,255] | 0.609 |
| Plato Pressure (cmH_2_O) | 19 [18,19] | 18 [17,18] | **0.032** |
| FiO2 (%) | 35 [35,50] | 35 [35,100] | 0.2559 |
| PEEP (cmH_2_O) | 5 [5,5] | 5 [5,5] | 0.346 |
|  |  |  |  |
| Heart Rate (bpm) | 93 [87,108] | 101 [80,119] | 0.666 |
| Mean Arterial Pressure  (mmHg) | 85 [72,100] | 100 [85,116] | 0.160 |
| Mean Pulmonary Artery Pressure  (mmHg) | 30 [22,31] | 22 [20,25] | 0.216 |
| Central Venous Pressure  (mmHg) | 10 [9,10] | 12 [7,12] | 1.000 |
| Pulmonary Artery Occlusion Pressure  (mmHg) | 12 [12,13] | 10 [9,16] | 1.000 |
| Cardiac Output (l/min) | 4.1 [3.7,4.8] | 5.5 [3.6,8.4] | 0.079 |
|  |  |  |  |
| Temperature (^o^C) | 37.5 [36.7,38.1] | 37.9 [36.6,38] | 0.361 |
| PAC SvO2 (%) | 50 [48,58] | 83 [83,94] | **<0.001** |
| pHa | 7.39 [7.35,7.40,] | 7.41 [7.20,7.42] | 0.882 |
| PaO2 (mmHg) | 84.3 [81.4,116] | 116 [101.6,298] | 0.1823 |
| PaCO2 (mmHg) | 40.0 [37.5,45.6] | 40.3 [40.0,42.4] | 0.488 |
| PvO2 (mmHg) | 31.9 [30.8,33.9] | 75.1 [56.4,100] | **0.021** |
| BE | -0.1 [-6.1],0.8] | 1.0 [-10,1.4] | 0.712 |
| SaO2 (%) | 94.4 [94.4,98.4] | 98.2 [97.8,100.0] | 0.087 |
| Lactate (mg/dl) | 6 [6,6] | 6 [6,8] | 0.565 |
| Hemoglobin (mg/dl) | 8.7 [8.4,8.8] | 8.8 [8.3,8.9] | 0.402 |

Table 2s: Comparison between stages 3 (S3) and 4 (S4). Variables without (Gas Flow OFF) and with ECMO support (Gas Flow ON). PAC SvO2 denotes venous oxygen saturation acquired in the Pulmonary Arterial Catheter; PaO2 and PvO2 denotes arterial and venous oxygen partial pressure, respectively; PaCO2 denotes arterial carbon dioxide partial pressure; SaO2 denotes arterial oxygen saturation and BE denotes Base Excess.

Figure 5s (A):PvO2=Venous Oxygen partial pressure. PvO2 significantly increased after initiation of ECMO support.

Figure 5s (B): PAPm=Mean Pulmonary Artery Pressure. PAPm did not change after initiation of ECMO support during bilateral ventilation and no previous hypoxemia.

Figure 5s (C): Left/Right Lung perfusion before and after initiation of ECMO support during bilateral ventilation. ECMO support by itself did not promote any variation on lung perfusion.

Figure 5s (D): Anterior/Posterior lung perfusion before and after initiation of ECMO support during bilateral ventilation. ECMO support by itself did not promote any variation on lung perfusion.

Comparison: Stage 3 -> Stage 5

Objective: To evaluate whether the ECMO circuit connected to the animal could induce changes in pulmonary perfusion or attenuate variations in pulmonary perfusion detected by EIT which could hinder the results obtained in later stages, before and after induction of unilateral pulmonary collapse.

|  | **S3-Bilateral Ventilation**  **ECMO + Gas Flow OFF** | **S5-Unilateral Ventilation**  **ECMO + Gas Flow OFF** | **P Value** |
| --- | --- | --- | --- |
| Respiratory Rate (ipm) | 22 [20,25] | 35 [35,35] | **0.019** |
| Tidal Volume (ml) | 240 [238,250] | 160 [152,175] | **0.023** |
| Plato Pressure (cmH_2_O) | 19 [18,19] | 24 [19,31] | 0.122 |
| FiO2 (%) | 35 [35,50] | 30 [25,35] | 0.355 |
| PEEP (cmH_2_O) | 5 [5,5] | 5 [5,5] | 0.346 |
|  |  |  |  |
| Heart Rate (bpm) | 93 [87,108] | 122 [114,134] | **0.025** |
| Mean Arterial Pressure  (mmHg) | 85 [72,100] | 105 [101,109] | 0.254 |
| Mean Pulmonary Artery Pressure  (mmHg) | 30 [22,31] | 37 [33,37] | **0.008** |
| Central Venous Pressure  (mmHg) | 10 [9,10] | 13 [9,13] | 0.426 |
| Pulmonary Artery Occlusion Pressure  (mmHg) | 12 [12,13] | 13 [12,13] | 0.108 |
| Cardiac Output (l/min) | 4.1 [3.7,4.8] | 4.6 [4.3,5.7] | 0.199 |
|  |  |  |  |
| Temperature (^o^C) | 37.5 [36.7,38.1] | 38 [37.2,38.2] | 0.194 |
| PAC SvO2 (%) | 50 [48,58] | 45 [43,46] | 0.410 |
| pHa | 7.39 [7.35,7.40,] | 7.39 [7.17,7.39] | 0.364 |
| PaO2 (mmHg) | 84.3 [81.4,116] | 61.8 [56.7,68] | 0.078 |
| PaCO2 (mmHg) | 40.0 [37.5,45.6] | 43.3 [38.5,47.1] | 0.153 |
| PvO2 (mmHg) | 31.9 [30.8,33.9] | 29.0 [25.3,30.4] | 0.084 |
| BE | -0.1 [-6.1],0.8] | -0.2 [-10.4,1.3] | 0.629 |
| SaO2 (%) | 94.4 [94.4,98.4] | 85.7[84.9,88.8] | 0.006 |
| Lactate (mg/dl) | 6 [6,6] | 8 [5,14] | 0.226 |
| Hemoglobin (mg/dl) | 8.7 [8.4,8.8] | 8.9 [8.7,9.7] | 0.198 |

Table 3s: Comparison between stages 3 (S3) and 5 (S5). Bilateral ventilation (S3) and Unilateral Ventilation (S5) after blood circulation through ECMO circuit. PAC SvO2 denotes venous oxygen saturation acquired in the Pulmonary Arterial Catheter; PaO2 and PvO2 denotes arterial and venous oxygen partial pressure, respectively; PaCO2 denotes arterial carbon dioxide partial pressure; SaO2 denotes arterial oxygen saturation and BE denotes Base Excess.

Figure 6s (A): Left lung ventilation before and after selective intubation and left lung collapse with blood flow through ECMO circuit. There was a significant reduction in left lung ventilation

Figure 6s (B): PvO2=Venous Oxygen partial pressure. PvO2 remained stable after selective lung ventilation and left lung collapse with blood flow through ECMO circuit.

Figure 6s (C): Left lung perfusion before and after selective intubation and left lung collapse with blood flow through ECMO circuit. Blood flow through ECMO did not affect the capacity of the EIT to detect the reduction in left lung perfusion

Comparison: Stage 5 -> Stage 6 -> Stage 7

Objective: To evaluate whether initiating support with ECMO and increased PvO2 could increase perfusion of the collapsed lung, which was reduced in response to hypoxic vasoconstriction,due to the collapse (Stage 5-> Stage -> 6) . Also, to evaluate if shutting down ECMO support would induce the opposite effect (Stage 6-> Stage -> 7). Data available on the original manuscript.

Comparison: Stage 8 -> Stage 9 -> Stage 10

Objective: Similar to the previous comparison, this one aims to evaluate whether starting support with ECMO and increased PvO2 could increase perfusion of the collapsed lung that was reduced in response to hypoxic vasoconstriction. However, here, we sought a model closer to the clinical picture of ARDS via induction of posterior collapse by surfactant depletion. Also, to evaluate if shutting down ECMO support would induce the opposite effect (Stage 9-> Stage -> 10). Data available on the original manuscript.
